# The immune response of young turkeys to haemorrhagic enteritis virus infection at different levels and sources of methionine in the diet

**DOI:** 10.1186/s12917-019-2138-8

**Published:** 2019-11-01

**Authors:** Bartłomiej Tykałowski, Marcin Śmiałek, Andrzej Koncicki, Katarzyna Ognik, Zenon Zduńczyk, Jan Jankowski

**Affiliations:** 1 Department of Poultry Diseases, Faculty of Veterinary Medicine, University of Warmia and Mazury, Oczapowskiego 13, 10-719 Olsztyn, Poland; 2 Department of Biochemistry and Toxicology, Faculty of Biology, Animal Sciences and Bioeconomy, University of Life Science in Lublin, Akademicka 13, 20-950 Lublin, Poland; 3 Institute of Animal Reproduction and Food Research of the Polish Academy of Sciences, Division of Food Science, Tuwima 10, 10-748 Olsztyn, Poland; 4 Department of Poultry Science, Faculty of Animal Bioengineering, University of Warmia and Mazury, Oczapowskiego 5, 10-719 Olsztyn, Poland

**Keywords:** Methionine, Haemorrhagic enteritis virus, Immune response, Turkeys

## Abstract

**Background:**

Haemorrhagic enteritis (HE) of turkeys was first described in 1937 in the USA, while in Poland it was first diagnosed in 1987. Polish haemorrhagic enteritis virus (HEV) isolates are usually low pathogenic and trigger a subclinical disease. Unfortunately, even the low- pathogenic HEV strains cause severe immunosuppression leading to secondary bacterial infections and huge economic losses. The objective of this study was to evaluate if the influence of Met on HEV infected turkeys immune response can be differentiated by both its level and source. Met is one of the amino acids that not only play a nutritional role but also participate in and regulate key metabolic pathways and immune response. In our study, the birds were assigned to 4 dietary treatments which differed in Met levels (0.55 and 0.78% in weeks 1–4 of age and 0.45 and 0.65% in weeks 5–8 of age, respectively) and sources (DL-methionine (DLM) or DL-methionine hydroxy analogue (MHA)).

**Results:**

The HEV added the percentage of CD4^+^ cells and decreased the percentage of IgM^+^ cells in the blood, spleen and caecal tonsils (CTs) of turkeys. In addition, it increased the percentage of CD4^+^CD25^+^ cells in blood, and interleukin-6 (IL-6) level in plasma. The higher dose of Met led to a significant decrease in the percentages of CD4^+^, CD8^+^ and CD4^+^IL-6^+^ cell subpopulations in the blood of HEV-infected and uninfected turkeys and to an increase in the percentage of IgM^+^ B cells in CTs. Turkeys administered feeds with an increased Met content displayed a decrease in plasma IL-6 levels and an increase in plasma IgA levels.

**Conclusions:**

The results of this study indicate that HEV infection impairs the immune function in turkeys. Met content in the feed has a moderate effect on the immune response in HEV-infected turkeys. The source of this amino acid appears not be as important as its dose, because value of the analysed parameters did not differ significantly between turkeys receiving feeds with DLM or MHA. In the uninfected turkeys, the higher by 40% (than recommended by NRC) level of Met in the feeds had a positive effect on humoral immunity parameters.

## Background

Haemorrhagic enteritis (HE) was first described in turkeys in 1937 by Pomeroy and Fenstermacher at Minnesota, USA [5]. In Poland, it was first diagnosed in 1987 [16]. Haemorrhagic enteritis virus (HEV) belongs to the family *Adenoviridae*, genus Siadenovirus. Under field conditions, susceptible to HEV infection are mainly turkeys over 4–6 weeks of life, whereas disease develops most often in 7–9 week old birds. HEV infection is usually asymptomatic. It may, however, proceed with depression, sanguineous excreta, and mortality rate approximating 80% only in the case of infection with highly pathogenic strains [5, 16, 40]. The Polish HEV isolates are usually low-pathogenic and induce subclinical infections, but severely impair the humoral and cellular reactivity of the immune system. At the acute stage of the disease, the percentage of B lymphocytes decreases significantly in blood and many immune organs, and the balance between percentage of CD4^+^ and CD8^+^ T lymphocytes is upset [40]. HEV has been shown to infect macrophages and reduce their functional capabilities. HEV-infected macrophages may undergo necrosis and apoptosis [28]. This may enhance the susceptibility to bacterial (usually *E. coli* and *Ornithobacterium rhinotracheale* (ORT)) and other viral infections in HEV-infected turkeys [5, 16, 17, 27].. An increased mortality rate due to superinfections with *E. coli* bacteria is usually observed between week 2 and 4 of life after infection with a low-pathogenic HE virus and may range from 2.5 to 6.5% weekly [24]. Considering the frequent exposure of turkeys to HEV infections and, consequently, their suppressed immunity, any actions aimed at aiding defence mechanisms in the course of the infection should be deemed highly desirable and valuable. One of the means to improve the health status of turkeys in commercial production is to supplement their feed mixtures with substances exhibiting immunostimulating effects. This is expected to alleviate negative outcomes of HEV and improve the birds health status and resultantly (by decreasing amounts of antibiotics used) to improve turkey production effectiveness and quality of poultry products. The substances with proved, positive impact on the immune system of poultry and their body weight gains include, i.a., vitamins, probiotics, essential oils, herbs, nucleotides, and amino acids [4, 18, 19].

Methionine (Met) is the first limiting amino acid in feeds of poultry because the Met content of natural feed ingredients is generally low (0.3–0.4%), below the requirements of fast-growing chickens and turkeys [12, 29]. Dietary Met content should be higher particularly in the first month of rearing, at 0.55% or even 0.70%, according to the recommendations of NRC [23] and British United Turkeys [2], respectively. Therefore, commercial feeds are usually supplemented with feed-grade Met, i.e. DL-methionine (DLM) or DL-methionine hydroxy analogue (MHA) [43]. Chemically, MHA is not an amino acid but a metabolic precursor of DLM and a very effective source of supplemental Met [7, 41]. Some studies have revealed MHA to be more effective in improving the antioxidant status of chickens and turkeys than DLM [25, 38, 42].

The results of recent research indicate that Met is one of the amino acids that not only play a nutritional role but also participate in and regulate key metabolic pathways [1, 11, 22] including those related to the immune system function [12, 13, 19]. One of the mechanisms proposed to explain Met interference in the immune system is the proliferation of T cells that are sensitive to intracellular variations in glutathione and cysteine levels, i.e. compounds which also participate in Met metabolism [8, 15]. The findings of other authors indicate that dietary Met can be a major contributor to the synthesis of immune system proteins, including antibodies such as IgA [44]. IgA is a major immunoglobulin synthesized locally and secreted onto the surface of mucous membranes of respiratory airways, gastrointestinal tract, reproductive system, and many other sites. It represents the main element of mucosal immune system. In blood serum, it circulates in the form of monomers in secretions with a dimer, a trimer or a tetramer. Mucosal IgA possesses a secretory component (SC) which promotes its adhesion to epithelial surface and provides protection from proteolytic degradation within cells [10].

Experimental evidence [3, 37] has shown that humoral stimulation and cellular immune responses were enhanced in broiler chickens administered feeds supplemented with Met in excess of NRC dietary recommendations [23]. In a study by Jankowski et al. [14], an increase in plasma IL-6 levels was noted in turkeys administered feeds with a higher Met content. In other experiment on young turkeys, the higher dietary Met content elevated IgA concentration and decreased IL-6 plasma level [45]. IL-6 is a multifunctional cytokine that plays a major role in regulating immune responses, acute phase reactions and haematopoiesis. It is a pro-inflammatory cytokine in both birds and mammals and is produced early after infection as part of the induced innate immune response. IL-6 plays a pivotal role during the transition from innate to acquired immunity, and is also involved in the regulation of metabolic, regenerative, and neural processes. This cytokine is produced by many different cell types (monocytes, macrophages, lymphocytes, fibroblasts, endothelial cells and chondrocytes) and acts on T cells, B cells, hepatocytes, haematopoietic progenitor cells and cells of the central nervous system [30, 31]. In addition, IL-6 plays a key role in the development of inflammatory lesions in intestinal mucosa of HEV-infected turkeys [27].

The available literature provides no information on the effects of Met on the immune system functioning in turkeys infected with a pathogen with a strong immunosuppressive activity. One of the objectives of this study was to evaluate if the influence of Met on the immune system functioning can be differentiated by both its level and source. Additionally, we have compared the immune responses of healthy and HEV- infected turkeys, administered feeds (1–8 weeks of age) containing two sources of Met (DLM and MHA) at two inclusion levels: recommended by NRC [23] (DLM_L_ and MHA_L_ groups) and approximately 40% higher than that recommended by NRC (DLM_H_ and MHA_H_ groups).

## Results

The average body weight (BW) of 56-day-old uninfected and HEV-infected turkeys ranged from 3.94 to 4.06 kg, and from 3.87 to 4.02 kg, respectively. No significant differences in BW were found between HEV-infected and uninfected turkeys administered feeds supplemented with DLM or MHA. From the day of experimental infection (42 days of age) until the end of the experiment (56 days of age), no mortality was recorded in the infected and uninfected groups.

Tables 1, 2 and 3 show the percentages of CD4^+^, CD4^+^CD25^+^, CD8^+^, CD4^+^CD8^+^ T cell subpopulations and IgM^+^ B cell subpopulation within the mononuclear cells isolated from the blood, spleen and caecal tonsils (CTs) of 47-day-old uninfected (0) and HEV- infected (HE) turkeys from groups DLM_L_, MHA_L_, DLM_H_ and MHA_H_. The percentages of the analysed T cell and B cell subpopulations in the blood (Table 1), spleen (Table 2) and CTs (Table 3) of turkeys were most influenced by HEV infection, followed by dietary Met levels (which exerted a weaker effect on the analysed parameters) and Met sources (which exerted a minor effect). In the acute phase of infection (first 5 days post inoculation (p.i.)), the HEV-infected turkeys displayed a significant increase in the percentage of CD4^+^ cells and a decrease in the percentage of IgM^+^ B cells in blood (both *P* < 0.001), spleen (both P < 0.001) and CTs (*P* = 0.005 and P < 0.001, respectively), compared with the uninfected birds. The higher dose of Met, regardless of its source, led to a significant decrease in the percentages of CD4^+^ cell (*P* = 0.009) and CD8^+^ cell (*P* = 0.027) subpopulations in the blood of HEV-infected and uninfected turkeys (Table 1), and an increase in the percentage of IgM^+^ B cells (*P* = 0.015) in CTs (Table 3).

**Table 1:** Percentages of peripheral blood T cell and B cell subpopulations in turkeys administered feeds with different Met sources and content (*n* = 7)

	CD4^+^	CD4^+^CD25^+^	CD8^+^	CD4^+^CD8^+^	IgM^+^
Group^1^					
0-DLM_L_	13.8	0.39	1.98	0.35	8.43
0-MHA_L_	14.8	0.39	2.08	0.39	8.24
0-DLM_H_	11.7	0.37	1.49	0.30	7.66
0-MHA_H_	11.2	0.38	1.86	0.33	9.22
HE-DLM_L_	29.1	0.46	2.87	0.53	5.90
HE-MHA_L_	25.3	0.46	1.60	0.50	5.77
HE-DLM_H_	21.6	0.42	2.24	0.51	5.67
HE-MHA_H_	19.8	0.46	1.75	0.50	6.31
Infection					
0	12.87	0.382	1.854	0.341	8.39
HE	23.96	0.450	2.116	0.511	5.91
Met dosage					
Low	20.76	0.423	2.295	0.445	7.06
High	16.07	0.409	1.675	0.407	7.24
Met source					
DLM	19.06	0.410	1.987	0.419	6.94
MHA	17.77	0.422	1.983	0.433	7.36
SEM^2^	1.250	0.013	0.138	0.022	0.255
*p* – values					
Infection	< 0.001	0.011	0.333	< 0.001	< 0.001
Dose	0.009	0.581	0.027	0.319	0.572
Source	0.450	0.636	0.987	0.712	0.197
Interaction	nd^3^	nd	nd	nd	nd

^1^ Feeds administered to uninfected (0) and infected (HE) turkeys in weeks 1–4 and 5–8, contained DLM and the equivalent amount of MHA at two levels: low (_L_) - 0.55, 0.45, and high (_H_) - 0.78, 0.65, respectively [% as fed]

^2^SEM- standard error of the mean

^3^nd – not detected: no significant infection x dose, infection x source, dose x source or infection x dose x source interactions

**Table 2:** Percentages of T cell and B cell subpopulations in the spleen in turkeys administered feeds with different Met sources and content (*n* = 7)

	CD4^+^	CD4^+^CD25^+^	CD8^+^	CD4^+^CD8^+^	IgM^+^
Group^1^					
0-DLM_L_	33.3	1.55	37.9	2.00	18.2
0-MHA_L_	35.3	1.65	33.5	2.46	18.6
0-DLM_H_	33.1	1.73	36.4	2.12	20.8
0-MHA_H_	29.3	1.75	37.3	2.15	21.8
HE-DLM_L_	51.4	1.67	25.0	1.88	15.8
HE-MHA_L_	55.9	1.61	24.7	2.09	14.9
HE-DLM_H_	53.8	1.83	19.1	2.24	16.7
HE-MHA_H_	50.4	1.76	21.4	2.12	14.7
Infection					
0	32.73	1.669	36.28	2.184	19.72
HE	52.85	1.716	22.56	2.083	15.53
Met dosage					
Low	43.95	1.620	30.29	2.108	16.89
High	41.63	1.765	28.55	2.159	18.36
Met source					
DLM	42.89	1.694	29.60	2.063	17.89
MHA	42.70	1.691	29.24	2.204	17.36
SEM^2^	1.860	0.046	1.477	0.109	0.558
*p* – values					
Infection	< 0.001	0.629	< 0.001	0.670	< 0.001
Dose	0.233	0.145	0.401	0.829	0.117
Source	0.921	0.972	0.864	0.552	0.568
Interactions	nd^3^	nd	nd	nd	nd

^1^ Feeds administered to uninfected (0) and infected (HE) turkeys in weeks 1–4 and 5–8, contained DLM and the equivalent amount of MHA at two levels: low (_L_) - 0.55, 0.45, and high (_H_) - 0.78, 0.65, respectively [% as fed]

^2^SEM- standard error of the mean

^3^nd – not detected: no significant infection x dose, infection x source, dose x source or infection x dose x source interactions

**Table 3:** Percentages of T cell and B cell subpopulations in the CTs in turkeys administered feeds with different Met sources and content (*n* = 7)

	CD4^+^	CD4^+^CD25^+^	CD8^+^	CD4^+^CD8^+^	IgM^+^
Group^1^					
0-DLM_L_	51.6	1.50	14.2	2.33	15.6
0-MHA_L_	53.9	1.61	14.0	1.83	14.5
0-DLM_H_	51.3	1.51	14.7	1.61	18.2
0-MHA_H_	46.8	1.87	15.1	1.85	18.1
HE-DLM_L_	55.6	1.44	15.9	1.77	11.1
HE-MHA_L_	61.3	1.55	14.6	2.38	10.9
HE-DLM_H_	57.0	1.46	13.4	3.05	12.5
HE-MHA_H_	54.1	1.64	15.1	2.23	12.7
Infection					
0	50.9	1.62	14.5	1.90	16.6
HE	57.0	1.52	14.8	2.36	11.8
Met dosage					
Low	55.6	1.52	14.7	2. 08	13.0
High	52.3	1.62	14.6	2.18	15.4
Met source					
DLM	53.9	1.48	14.6	2.19	14.3
MHA	54.0	1.67	14.7	2.07	14.0
SEM^2^	1.12	0.031	0.458	0.156	0.603
*p* – values					
Infection	0.005	0.060	0,805	0.149	< 0.001
Dose	0.110	0.062	0.895	0.738	0.015
Source	0.939	0.001	0.905	0.704	0.744
Interactions	nd^3^	nd	nd	nd	nd

^1^ Feeds administered to uninfected (0) and infected (HE) turkeys in weeks 1–4 and 5–8, contained DLM and the equivalent amount of MHA at two levels: low (_L_) - 0.55, 0.45, and high (_H_) - 0.78, 0.65, respectively [% as fed]

^2^SEM- standard error of the mean

^3^nd – not detected: no significant infection x dose, infection x source, dose x source or infection x dose x source interactions

Both the HEV experimental infection and increased dietary Met content contributed to a significant (*P* < 0.001) decrease in the blood percentage of CD4^+^ cells, synthesizing IL-6 in response to mitogenic stimulation under in vitro conditions. The HEV infection had a similar effect on IL-6 synthesis by CD4^+^ cells isolated from the spleen and CTs of turkeys (Table 4).

**Table 4:** The percentage of CD4^+^ cells isolated from blood, spleen and CTs, synthesizing IL-6 in response to mitogenic stimulation under in vitro conditions (*n* = 7)

	Blood	Spleen	CTs
Group^1^			
0-DLM_L_	1.67	0.88	0.58
0-MHA_L_	1.38	0.67	0.57
0-DLM_H_	1.09	0.68	0.62
0-MHA_H_	0.84	0.77	0.58
HE-DLM_L_	0.97	0.38	0.48
HE-MHA_L_	0.52	0.38	0.44
HE-DLM_H_	0.85	0.32	0.49
HE-MHA_H_	0.40	0.32	0.43
Infection			
0	1.247	0.751	0.59
HE	0.685	0.354	0.46
Met dosage			
Low	1.219	0.580	0.51
High	0.713	0.525	0.53
Met source			
DLM	1.064	0.567	0.54
MHA	0.868	0.538	0.50
SEM^2^	0.069	0.036	0.018
*p* – values			
Infection	< 0.001	< 0.001	< 0.001
Dose	< 0.001	0.097	0.661
Source	0.004	0.361	0.204
Interactions	nd^3^	nd	nd

^1^Feeds administered to uninfected (0) and infected (HE) turkeys in weeks 1–4 and 5–8, contained DLM and the equivalent amount of MHA at two levels low (_L_) - 0.55, 0.45, and high (_H_) - 0.78, 0.65, respectively [% as fed]

^2^SEM- standard error of the mean;

^3^nd – not detected: no significant infection x dose, infection x source, dose x source or infection x dose x source interactions

A significant (*P* < 0.001) increase in plasma IL-6 concentrations was noted in the HEV-infected turkeys, compared with the uninfected birds. Plasma IL-6 levels were lower in turkeys fed with the higher MHA dose (Table 5).

**Table 5:** Plasma IgA and IL-6 levels in turkeys (n = 7)

	IgA [μg/ml]	IL-6 [pg/ml]
Group^1^		
0-DLM_L_	0.176	0.141^b^
0-MHA_L_	0.096	0.161^b^
0-DLM_H_	0.159	0.121^bc^
0-MHA_H_	0.278	0.118^bc^
HE-DLM_L_	0.178	0.385^a^
HE-MHA_L_	0.086	0.101^bc^
HE-DLM_H_	0.101	0.201^b^
HE-MHA_H_	0.103	0.070^c^
Infection		
0	0.177	0.135
HE	0.117	0.189
Met dosage		
Low	0.134	0.197
High	0.160	0.127
Met source		
DLM	0.153	0.212
MHA	0.141	0.113
SEM^2^	0.012	0.017
*p* – values		
Infection	< 0.001	< 0.001
Dose	0.068	< 0.001
Source	0.414	< 0.001
Interactions	nd^3^	d^4^

^1^ Feeds administered to uninfected (0) and infected (HE) turkeys in weeks 1–4 and 5–8, contained DLM and the equivalent amount of MHA at two levels low (_L_) - 0.55, 0.45, and high (_H_) - 0.78, 0.65, respectively [% as fed]

^2^SEM- standard error of the mean

^3^nd – not detected: no significant infection x dose, infection x source, dose x source or infection x dose x source interactions

^4^d- detected: Significant interactions between the experimental factors; higher dose of DLM and both doses of MHA decreased plasma IL-6 levels in HEV-infected turkeys

^a-c^ values differ significantly

Anti-ND antibody titres were not significantly affected by dietary Met sources or levels, but they were significantly (*P* = 0.004) lower in the HEV-infected turkeys than in the uninfected birds (Table 6). Average vaccine-induced anti-ORT antibody titres were significantly (P < 0.001) higher at 56 days of age in the HEV-infected turkeys than in the uninfected birds.

**Table 6:** Vaccine-induced antibody titres in the blood serum of 56-day-old turkeys (*n* = 23)

	ORT	NDV
Group^1^		
0-DLM_L_	12879^b^	236
0-MHA_L_	11116^b^	768
0-DLM_H_	15022^b^	443
0-MHA_H_	14571^b^	376
HE-DLM_L_	23072^a^	69
HE-MHA_L_	17976^ab^	150
HE-DLM_H_	15849^b^	117
HE-MHA_H_	19120^b^	139
Infection		
0	13,397	441
HE	19,004	119
Met dosage		
Low	14,581	358
High	15,611	324
Met source		
DLM	15,620	265
MHA	14,572	421
SEM^2^	687	185
*p* – values		
Infection	< 0.001	0.004
Dose	0.932	0.746
Source	0.473	0.215
Interactions	0.039^3^	nd^4^

^1^Feeds administered to uninfected (0) and infected (HE) turkeys in weeks 1–4 and 5–8, contained DLM and the equivalent amount of MHA at two levels: low (_L_) - 0.55, 0.45, and high (_H_) - 0.78, 0.65, respectively [% as fed]

^2^SEM- standard error of the mean

^3^Significant interactions between the experimental factors; HEV added anti-ORT antibody titres, particularly in turkeys fed feeds with lower DLM content

^4^nd – not detected: no significant infection x dose, infection x source, dose x source or infection x dose x source interactions

## Discussion

No deterioration of production performance was observed in our study for the HEV-infected turkeys administered feeds supplemented with DLM or MHA. The Polish isolate of HEV used in the experiment is characterised by low pathogenicity and, in the absence of secondary bacterial infections, it does not increase birds mortality or significant changes in BW. However, even low-virulence strains that cause subclinical infection can result in immunosuppression [16, 17, 40].

Results of our study indicate that the HEV infection strongly influences the immune system of turkeys, which leads to a significant increase in the percentage of CD4^+^ cells and a decrease in the percentage of IgM^+^ B cells in their blood, spleen, and CTs. Present results corroborate previous findings [17, 36]. There is a scarcity of studies investigating the effect of HEV on the percentages of the analysed cell subsets in CTs. Regulatory T cells (T_reg_), a subset of T cells identified by the co-expression of CD4 and CD25, suppress immune responses to self-antigens and prevent autoimmune diseases. There is emerging evidence to suggest that regulatory T cells control immune responses to bacteria, viruses, parasites and fungi [21]. Regulatory T cells have been identified in several species, including humans, dogs, cats, pigs, cows, sheep, horses, chickens and turkeys [6, 32, 33]. The mechanism of the suppressive function of natural and inducible regulatory T cells is still debated, but in different model systems, suppressive activity has been shown to be mediated either through secretion of immunosuppressive cytokines or through cell–cell contact [21].

Many studies have shown that pathogens, in particular those that cause chronic infections or are associated with immunosuppression, induce the production of regulatory cytokines IL-10 and TGF-β [20]. Turkey thymic CD4^+^CD25^+^ cells have approximately 158-fold higher IL-10 mRNA, 7-fold higher TGF-β, 24-fold higher CTLA-4 and 11-fold higher LAG-3 mRNA amounts than thymic CD4^+^CD25^−^ cells [33].

There is a scarcity of studies investigating the role of regulatory T cells in the course of HEV infection in turkeys. In the current study, the percentage of CD4^+^CD25^+^ cells increased significantly in the blood of HEV-infected turkeys on day 5 p.i. A significant increase in the percentage of CD4^+^CD25^+^ cells was also noted in the CTs of the turkeys administered MHA-supplemented feeds relative to the birds fed DLM-supplemented feed mixtures. Our results and the findings of other authors [9, 28, 36] indicate that HEV impairs immune function in infected turkeys, leading to strong immunosuppression. After exposure, HEV may replicate in B lymphocytes located in the intestines and the bursa of Fabricius, or it may travel directly to the spleen through peripheral blood infecting numerous macrophages and B cells, and replicate efficiently [35]. The infection of target cells in the spleen leads to the influx of macrophages and CD4^+^ T cells in the white pulp, resulting in hyperplasia. The activation of macrophages by HEV leads to the production of cytokines such as IL-6, tumour necrosis factor (TNF) and interferon (IFN) type I and II [28]. In the acute phase of the disease, the depletion of IgM^+^ B cells occurs in the blood and spleen of birds [17, 36]. Virus-induced cell necrosis and apoptosis may compromise the antigen-presenting function and possibly other functions of macrophages and B cells. In the present study, the higher dietary Met level induced a lesser decrease in the percentage of IgM^+^ B cells in HEV-infected turkeys, particularly in their CTs.

Results of our study demonstrate that the higher supplementation levels of Met, used in the form of DLM and MHA, reduced the blood level of IL-6 in both the HEV-infected and uninfected turkeys. IL-6 plays a major role in regulating immune responses and acute phase reactions [30, 31]. It is a pro-inflammatory cytokine and is produced early (2–3 days p.i) after HEV infection as part of the induced innate immune response. It appears that in our study, cells other than CD4^+^ lymphocytes, activated by HEV, secreted IL-6 and contributed to an increase in the plasma concentrations of this interleukin. Earlier investigations conducted by other authors have demonstrated high IL-6 and TNF level at the peak of the acute phase in HEV-infected turkeys. Cytokines may play a protective as well as a destructive role. While a massive release of proinflammatory cytokines may lead to systemic shock associated with haemorrhagic enteritis and death [27].

Immunosuppression induced by HEV infection may impair post-vaccination immunity after the administration of live vaccines, which was observed in the current study in turkeys vaccinated against ND. Usually, once-vaccinated turkeys do not develop high levels of serum antibodies as detected in the ELISA test. Our findings correspond to the observations of other authors [9]. In turkeys that had been vaccinated twice against ORT with an inactivated vaccine, vaccine-induced antibody titres were significantly higher in the serum of 56-day-old birds administered feeds with increased Met content, which corroborates the findings of Kubińska et al. [19]. Average plasma IgA levels were also higher in turkeys receiving higher Met concentrations. Our findings are consistent with the results of other studies which show that dietary Met can be a major contributor to the synthesis of immune system proteins, including IgA [12, 44].

Interesting results of serological analyses were obtained in the HEV-infected turkeys that were revaccinated against ORT at 49 days of age (7 days p.i.). Over that period, the number of HEV Hexon gene copies, determined in the spleen by qPCR, is higher than 3 and 14 days p.i. (Tykałowski B, unpublished data). Despite this fact, average vaccine-induced anti-ORT antibody titres were higher at 56 days of age in the HEV-infected turkeys than in the uninfected birds. The increase in anti-ORT antibody titres could have resulted from the activation of the immune system of the HEV-infected turkeys. Similar results were reported by Rautenschlein and Sharma [26]. In the cited study, 5- to 6-week-old turkeys were simultaneously vaccinated with attenuated NDV-B1 ELD_50_ = 10^5^ /bird intraocular) and cell culture-adapted HEV (HEVp30) at TCID_50_ = 10^4^ /bird per os. At 14 days post vaccination, the anti-NDV antibody response was significantly enhanced (*P* < 0.05) in HEVp30 + NDV-vaccinated turkeys in comparison with the single-inoculated birds. At 21 days post vaccination, anti-HEV and anti-NDV antibody responses were similar in all vaccinated groups. However, the combination of HEVp30 and NDV-B1 enhanced the rate of apoptosis in splenic cells. However, the mechanism of the enhancement of anti-NDV antibodies remains unknown.

## Conclusions

The results of this study indicate that HEV infection impairs immune function in turkeys. Depending on its content in the feed, methionine used in our study moderately affected the immune response of HEV-infected turkeys by alleviating infection outcomes. The source of this exogenous amino acid is not as important as its dose is, because values of the analysed parameters did not differ significantly between turkeys administered feeds with DLM or MHA. In the uninfected turkeys, the higher by 40% level of methionine in the feed (than that recommended by NRC) had a positive effect on humoral immunity parameters, causing an increase both in the percentage of B lymphocytes in blood, spleen and CTs, and in plasma level of IgA.

## Methods

### Birds, management and feeds

The study was conducted on 240 day-old female Hybrid Converter turke**y** poults purchased from a local hatchery (Grelavi S.A., Ketrzyn, Poland) and kept in isolated pens in the Pavilion of Avian Experimental Infections of the Department of Avian Diseases of the University of Warmia and Mazury in Olsztyn. The facility conforms to the relevant biosafety level (BSL-3). The birds were randomly assigned to 4 dietary treatments (60 birds per treatment). Each treatment was further divided into two groups (kept in 2 separate isolated pens, 30 birds per group). The total number of birds in each group was adapted to the size of experimental boxes. The dietary treatments differed in the level (low - “_L_”or high – “_H_”) and the source (DLM or MHA) of Met in feed. In each dietary treatment (DLM_L_, MHA_L_, DLM_H_ and MHA_H_, respectively), one group was inoculated with HEV and the other group served as uninfected control. The experiment layout is presented in Table 7.

**Table 7:** Experiment layout

Dietary treatment^1^ (*n* = 60)	Group (*n* = 30)	Days of age					
		10	28	42 (experimental infection)	47	49	56
DLM_L_	0-DLM_L_	Vaccination against ND	Vaccination against ORT	0^2^	Collection of samples of blood and organs from 7 turkeys per group for immunological (CD4^+^, CD8^+^, CD4^+^CD8^+^, CD4^+^CD25^+^, CD4^+^IL-6^+^,IgM^+^) and biochemical analyses (IgA, IL-6)	Vaccination against ORT	Collection of blood samples from 23 turkeys per group for serological analyses (antibody against ORT and NDV)
	HE-DLM_L_			+ ^3^			
MHA_L_	0-MHA_L_			0			
	HE-MHA_L_			+			
DLM_H_	0-DLM_H_			0			
	HE-DLM_H_			+			
MHA_H_	0-MHA_H_			0			
	HE-MHA_H_			+			

^1^ Feeds administered to turkeys in weeks 1–4 and 5–8, contained DLM and the equivalent amount of MHA at two levels: low (_L_) - 0.55, 0.45, and high (_H_) - 0.78, 0.65, respectively [% as fed

^2^ 0 - uninfected turkeys (received 1 ml of sterile PBS into the crop)

^3^+ − turkeys inoculated with 1 ml of a suspension containing HEV at a dose of 10^4,3^ EID_50,_ administered into the crop

The temperature and lighting programs were consistent with the recommendations of Hendrix Genetics Ltd. (Canada) for Hybrid Converter turkeys. The birds had free access to feed and water. All experimental procedures were consistent with permission No. 45/2013 of the Local Ethics Committee for Animal Experiments in Olsztyn (Poland). NRC [23] recommendations were adopted as the low dietary levels of DLM (Evonik Industries, Krefeld, Germany) and MHA (calcium salt of 2-hydroxy-4-(methyl) butanoic acid, Novus International, Inc., USA). The dietary Met content increased by 40% relative to NRC [23] recommendations was regarded as the high level which contributed to achieving good turkey performance in previous experiments [13, 18, 19].

The nutritional value of basal feeds prepared in two successive 4-week periods (Table 8) was calculated according to the Polish Feedstuff Analysis Tables [34]. The analytically verified total Met content of experimental feeds, i.e. the lower and higher dietary levels of Met (including the equivalent amount of MHA), was 0.55 and 0.78% in weeks 1–4 of age, respectively, and 0.45 and 0.65% in weeks 5–8 of age, respectively.

**Table 8:** Composition and nutrient concentrations in basal feeds

Item	Feeding period (weeks)	
	1–4	5–8
Ingredients as fed [g/kg]		
Wheat	49.09	49.26
Soybean meal	41.65	40.56
Soybean oil	2.02	2.90
Fish meal	3.00	–
Rapeseed meal	–	3.00
Sodium sulphate	0.15	0.15
Sodium chloride	0.11	0.15
Limestone	1.41	1.46
Monocalcium phosphate	1.61	1.52
L-Lysine HCl	0.38	0.41
L-Threonine	0.08	0.09
Vitamin-mineral premix^a^	0.50	0.50
Calculated nutritional value^b^		
Metabolizable energy [Kcal/kg]	2750	2850
Crude protein [%]	27.0	25.0
Arginine [%]	1.71	1.59
Lysine [%]	1.74	1.603
Methionine [%]	0.40	0.34
Methionine + cysteine [%]	0.84	0.76
Threonine [%]	1.05	0.98
Ca [%]	1.20	1.10
P [%]	0.81	0.74
Available P [%]	0.58	0.50
Sodium [%]	0.15	0.13

^a^0.5% of the premix provided per kg of diet: vitamin a (all trans-retinol acetate) – 15,000 IU, vitamin D_3_ (cholecalciferol) – 5000 IU, vitamin E (all-rac-α-tocopheryl acetate) - 100 mg, vitamin K_3−_4 mg, vitamin B_1−_5 mg, vitamin B_2−_15 mg, vitamin B_6−_6 mg, niacin - 100 mg, biotin - 0.35 mg, pantothenic acid - 32 mg, nicotinic acid −100 mg, folic acid - 4 mg, choline chloride - 700 mg, Mn - 100 mg, Zn - 80 mg, Fe - 60 mg, cu - 20 mg, I - 1.5 mg, se - 0.3 mg, Ca – 1.07 g

^b^Calculated according to the Polish Feedstuff Analysis Tables [34]

### Vaccination

Experimental turkeys fell under a standard vaccination program, practiced at commercial farms in Poland. At 10 days of age, all 240 turkeys were vaccinated against Newcastle disease virus (NDV). The vaccine (Nobilis® ND, Clone 30, MSD, USA) was administered in a dose recommended by the producer with a dropper at one drop (0.05 ml) per bird into one eye. At 28 and 49 days of age, turkeys were vaccinated against ORT by subcutaneous administration of 0.5 ml of Ornitin vaccine (ABIC, Poland).

### Experimental inoculation with HEV

At 42 days of age, turkeys from HE-DLM_L_, HE-MHA_L_, HE-DLM_H_, and HE-MHA_H_ groups were experimentally inoculated with 1 ml of a suspension containing HEV at a dose of 10^4,3^ EID_50_, administered into the crop with a probe [16]. In turn, turkeys from 0-DLM_L_, 0-MHA_L_, 0-DLM_H_, and 0-MHA_H_ group received 1 ml of PBS via the same route.

### Sample collection

Sample collection times and experiment design were presented in Table 7. All activities (vaccination, infection, sample collection) were performed between 7.00 and 8.00 a.m.

The immunological parameters were determined in blood, spleen and CTs. For this study, 7 turkeys at 47 days of life (representing average BW ± 10% per pen) were selected from each group, commonly accepted as the minimum number of turkeys with a unified genotype that ensures reliable, reproducible and statistically significant results without repeating the procedure due to high intra-group variability. Blood samples were collected from the wing vein into sterile test tubes (BD Vacutainer®, USA) with the K2EDTA anticoagulant for flow cytometry or with lithium heparin for biochemical analysis. Selected birds from each group were euthanized and their spleen and CTs were collected for mononuclear cell isolation and determination of the percentages of CD4^+^, CD8^+^, CD4^+^CD8^+^, CD4^+^CD25^+^, CD4^+^IL-6^+^ T cell subpopulations and IgM^+^ B cell subpopulation, by flow cytometry. Humanitarian euthanasia of the birds was performed with the use of a professional UNO Euthanasia Unit (UNOBV, Netherlands). The turkeys were placed in a chamber to which Carbogen (95% O2 + 5% CO2) was provided by the unit. After 1 min, Carbogen flow was stopped and 100% CO_2_ was introduced into the cage. In this way, the high concentration of O_2_ was slowly replaced by CO_2_. This relatively slow replacement of O_2_ by CO_2_ is responsible for stress reduction in the birds.

At 56 days of age, all turkeys were weighed, and blood samples were collected from 23 birds per treatment into sterile test tubes without the anticoagulant for serological analysis.

### Isolation of mononuclear cells and flow cytometry

Mononuclear cells from blood, spleen and CTs were isolated according to a previously described procedure [17, 39]. The cells were counted, and their viability was evaluated using the Vi-Cell XR cell counter (Beckman Coulter, USA).

### Determination of the percentages of selected T cell and B cell subpopulations in blood, spleen and CTs

Viable mononuclear cells (1 × 10^6^) were stained with fluorescein conjugated Mouse Anti-Chicken CD4-FITC (MCA2164F, Bio-Rad, UK), phycoerythrin conjugated Mouse Anti-Chicken CD8a-RPE (MCA2166PE, Bio-Rad, UK) and Alexa Fluor® 647 conjugated Human Anti-Chicken CD25 (HCA173A647, Bio-Rad, UK) or Alexa Fluor® 647- conjugated Negative Control Antibody (HCA052A647, Bio-Rad, UK). B cells were stained separately with FITC-conjugated Goat Anti-Chicken IgM (AAI27F, Bio-Rad, UK). The percentages of CD4^+^, CD4^+^CD25^+^, CD8^+^, CD4^+^CD8^+^ and IgM^+^ cells were determined in a FACSCanto II flow cytometer (BD, USA). Cell staining, acquisition and analysis of cytometric data were described elsewhere [18].

### Determination of the percentage of IL-6 synthesizing cells within the CD4^+^ subpopulation

Mononuclear cells isolated from blood, spleen and CTs were individually transferred at 2 × 10^6^ to 24-well plates (Corning, USA). Each well contained 2 ml of complete culture medium (RPMI-1640, 20 mM HEPES, 10% FBS, Antibiotic Antimycotic Solution; Sigma-Aldrich, Germany), 4 μl of Leukocyte Activation Cocktail with BD GolgiPlug™ (BD Pharmingen, USA) and 0.3 μg/ml of co-stimulatory Mouse Anti-Chicken CD28 monoclonal antibody (MCA5760, clone AV7, Bio-Rad, UK). Cells collected from each turkey were analysed in triplicate. The cells were incubated at a temperature of 40 °C (5% CO_2_) for 6 h. After incubation, 20 μl of 20 mM EDTA solution (Sigma-Aldrich, Germany) in PBS was added to each well. The contents of each well were individually transferred to test-tubes, and were rinsed twice with PBS supplemented with 1% FBS. The cells were stained with Mouse Anti-Chicken CD4-FITC monoclonal antibody (MCA2164F, clone 2–35, Biorad, UK). The samples were incubated on ice in darkness for 30 min, and were rinsed with PBS. The cells were fixed with 100 μl of Leucoperm reagent A (Biorad, UK), and were incubated at room temperature for 15 min. After incubation they were washed with PBS at 300 g for 5 min. Afterwards, they were suspended in 100 μl of permeabilization medium (Reagent B, Leucoperm, Biorad, UK), and 5 μl of Rabbit Anti-Chicken IL-6 antibody was added (AHP942Z, Biorad, UK). Thoroughly mixed samples were incubated at room temperature in darkness for 30 min. After incubation, the cells were washed with PBS. The cells were suspended in 100 μl of PBS, and 5 μl of secondary antibodies Sheep Anti Rabbit IgG:PE (STAR35A, Bio-Rad, UK) was added. The samples were incubated at room temperature in darkness for 30 min. After incubation, the cells were washed with PBS. Final cell pellets were suspended in 400 μl of PBS, and analysed with FACSCanto II flow cytometer (BD, USA).

A fluorescence minus one (FMO) control was prepared for each analysed sample. Primary Anti-Chicken IL-6 antibodies were not added to FMO controls. The cytometer setup and tracking beads (CST, BD, USA) were used to initialise photomultiplier tubes settings. Unstained and single-stained control cells for each fluorochrome were prepared and used to set up flow cytometry compensation.

### Biochemical and serological analyses

Immediately after collection, blood samples were centrifuged (15 min, 380 g, 4 °C) and the obtained plasma or serum was stored at − 20 °C until analysis. Plasma levels of IgA and IL-6 were determined with the use of the Bigenet UMV340 blood cell reader (Horiba, Kyoto, Japan) and kits for determining IL-6 (SEA079Ga, Wuhan USCN Business Co., China) and IgA (CSB-E11232Ch, Cusabio Biotech Co., China).

Vaccine-induced titres of serum antibody against ORT and ND were determined with the use of commercial ELISA kits (Idexx Laboratories, USA). The analytical procedure was consistent with the manufacturer’s recommendations. Absorbance was measured with the Elx 800 spectrophotometer (BioTek, USA). The results were analysed using the xChek 3.3 programme (Idexx Laboratories, USA).

### Statistical analysis

The results were analysed statistically by three-way analysis of variance (ANOVA) using STATISTICA software version 12.0 (StatSoft Inc., USA). The significance of differences between means was determined by the F-test and Duncan’s multiple range test. Data are presented as means ± SEM and the value of *p* < 0.05 was considered statistically significant. SEM was estimated by dividing the standard deviation by the square root of replication number.

## Data Availability

The datasets generated and/or analysed during the current study are not publicly available due to restrictions in the contract with the funding body but are available from the corresponding author on reasonable request.

## Abbreviations

BSL-3: biosafety level-3
BW: Body weight
CD: Cluster of differentiation
CST: Cytometer setup and tracking beads
CTLA-4: Cytotoxic T-lymphocyte associated protein-4
CTs: Caecal tonsils
DLM: DL-methionine
EDTA: Ethylenediaminetetraacetic acid
ELD_50_: Egg lethal dose
FMO: Fluorescence minus one
HE: Haemorrhagic enteritis
HEV: Haemorrhagic enteritis virus
IFN: Interferon
IgA: Immunoglobulin A
IgM: Immunoglobulin M
IL: Interleukin
LAG-3: Lymphocyte activation protein 3
Met: Methionine
MHA: DL-methionine hydroxy analogue
NDV: Newcastle disease virus
ORT: *Ornithobacterium rhinotracheale*
p.i.: Post inoculation
SC: Secretory component
SEM: Standard error of the mean
TCID_50_: Tissue culture infective dose
TGF-β: Transforming growth factor-β
TNF: Tumour necrosis factor
T_reg_: Regulatory T cells
